# Case Report of Plastic String Entanglement Mortality in a Breeding Oriental Reed Warbler

**DOI:** 10.1002/ece3.73009

**Published:** 2026-01-30

**Authors:** Haijie Zhang, Yufeng Liu, Yingying Wang, Gang Fu, Laikun Ma, Jianxin Dong

**Affiliations:** ^1^ Hebei Minzu Normal University Chengde China; ^2^ Institute of Geographical Sciences, Hebei Academy of Sciences Hebei Technology Innovation Center for Geographic Information Application Shijiazhuang China

**Keywords:** anthropogenic hazards, avian breeding, nesting material, oriental reed warbler, plastic products

## Abstract

Rapid economic development and urbanization have exerted considerable impacts on the environment and wildlife. Species that survive and establish populations in new environments benefit from abundant food resources and reduced predation pressure; however, they are also exposed to certain risks. Plastic production and use have increased markedly alongside population growth and urbanization, with large quantities of plastic products entering ecological environments. Whereas birds and other animals use them as nesting materials, plastic nesting materials pose toxicity and entanglement risks, affecting bird survival and reproduction. In addition, although numerous studies have reported cases of avian mortality due to entanglement, such studies have predominantly focused on medium to large species (mostly seabirds) and nestlings. This study documents a case observed on June 28, 2025, in the Baiyangdian Wetland, Hebei Province, China, where an adult breeding Oriental Reed Warbler parent bird died due to entanglement in plastic string present in its nesting material. The nest contained one unhatched egg, and another parent bird was observed maintaining vigilance near the nest. No subsequent egg‐laying was observed, suggesting that the deceased parent was likely female and that the breeding attempt failed. Subsequently, the nest status was photographed and documented. Based on local conditions, the plastic string probably originated from fishing equipment or reed‐weaving. This case directly demonstrates the hazards that plastic products pose to avian survival and reproduction.

## Graphical abstract

Plastic production and use have increased markedly, concomitantly with rapid urbanization and development. Whereas birds and other animals use them as nesting materials, they pose toxicity and entanglement risks, potentially affecting survival and reproduction. This study documents a case where an Oriental Reed Warbler parent bird died following entanglement in plastic string in its nesting material.

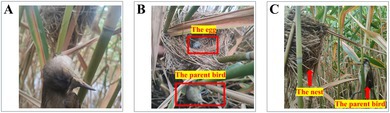

## 1 Introduction

With the progression of urbanization, direct and indirect human disturbance has emerged as key factors threatening wildlife survival (Almond et al. 2022). Human activities such as hunting and netting directly reduce bird populations (Benitez‐Lopez et al. 2017; Liang et al. 2023, 2025). Meanwhile, the indirect negative impacts of anthropogenic activities and disturbance cannot be overlooked, including habitat fragmentation, which degrades bird habitat quality (Haddad et al. 2015; Marcolin et al. 2021), urban heat island effects, which decrease urban bird diversity (Cai et al. 2023), and improper disposal of hazardous substances, which pollute bird habitats (Richard et al. 2021). However, human activities do not always negatively impact birds; for instance, urban environments reduce predation probability for certain bird species (Eötvös et al. 2018) and urban resources provide food sources for some overwintering bird species (Van Doren et al. 2021).

Breeding constitutes the most important component of avian life history, and it is directly related to population continuity and population size fluctuations (Sibly et al. 2012; Kaliński et al. 2019). Under natural conditions, multiple factors, including predation (Maziarz et al. 2016), brood parasitism (Stevens 2013; Zhang et al. 2023), extreme weather and natural disasters (Solís et al. 2024; Zhang et al. 2024), and parental personalities (Li et al. 2024) influence avian reproductive success. With an increase in the intensity of human activities globally, anthropogenic factors, such as light and noise pollution (Senzaki et al. 2020), toxic chemicals (Richard et al. 2021), and artificial nesting materials (Ryan 2018), increasingly interfere with avian breeding success.

Plastic, as an artificial material, is used extensively globally due to its low cost, light weight, long lifespan, and excellent barrier properties (Ryan 2018). However, large quantities of plastic waste are discarded carelessly in the environment and are difficult to biodegrade, triggering garbage accumulation and microplastic pollution challenges globally (Cottom et al. 2024; Zhang et al. 2021). Avian ingestion of environmental plastics may reduce food intake (Pierce et al. 2004) or cause reproductive system diseases (Roman et al. 2019), potentially resulting in individual mortality or reproductive failure. Some birds incorporate plastic into their nesting materials (Chen et al. 2024). For example, the Red‐backed Shrike ( *Lanius collurio* ) inhabiting agricultural landscapes utilizes plastic debris from the environment, particularly plastic string fibers, as nesting material (Kwieciński et al. 2024). This may lead to entanglement injuries and even death in birds, consequently affecting their reproductive success (Townsend and Barker 2014). A similar risk has been documented in the White Stork ( *Ciconia ciconia* ), which uses plastic string as a material to improve the nest environment during nest‐building. However, such behavior has led to numerous nestlings becoming entangled in the plastic string, resulting in deteriorating health and even death (Kwieciński et al. 2006).

The Oriental Reed Warbler ( *Acrocephalus orientalis* ) belongs to the order Passeriformes and family Acrocephalidae. It is a small bird species approximately 18 cm in length that primarily inhabits and breeds in reed wetlands (Liu and Chen 2021). Previous studies have focused mainly on reproductive strategies such as coevolution with brood‐parasitic birds such as the Common Cuckoo ( *Cuculus canorus* ) (Li et al. 2015; Ma and Liang 2021; Ma et al. 2024; Wang et al. 2025, 2023). This study documents a case of a breeding adult Oriental Reed Warbler that died due to entanglement in plastic string present in its nesting material.

## 2 Methods and Results

### 2.1 Study Site Overview

The study area is located in the Baiyangdian Wetland, Hebei Province, China (38°43′–39°10′ N, 115°38′–116°19′ E, approximately 10 m above sea level), within a temperate semi‐arid continental monsoon climate zone. The study site has an annual average temperature of 12.2°C, with an extreme maximum temperature of 40.7°C and an extreme minimum temperature of −26.7°C (Wang et al. 2025). With urbanization and tourism development, fishing activities by local residents and tourists have increased in frequency. Fishing tools include nets and fishing lines, and these tools are predominantly made of plastic. Additionally, the local area produces reed products that contain large quantities of plastic rope; consequently, numerous discarded plastic products remain in the natural environment.

### 2.2 Documentation of Entanglement Mortality Case

**FIGURE 1:**
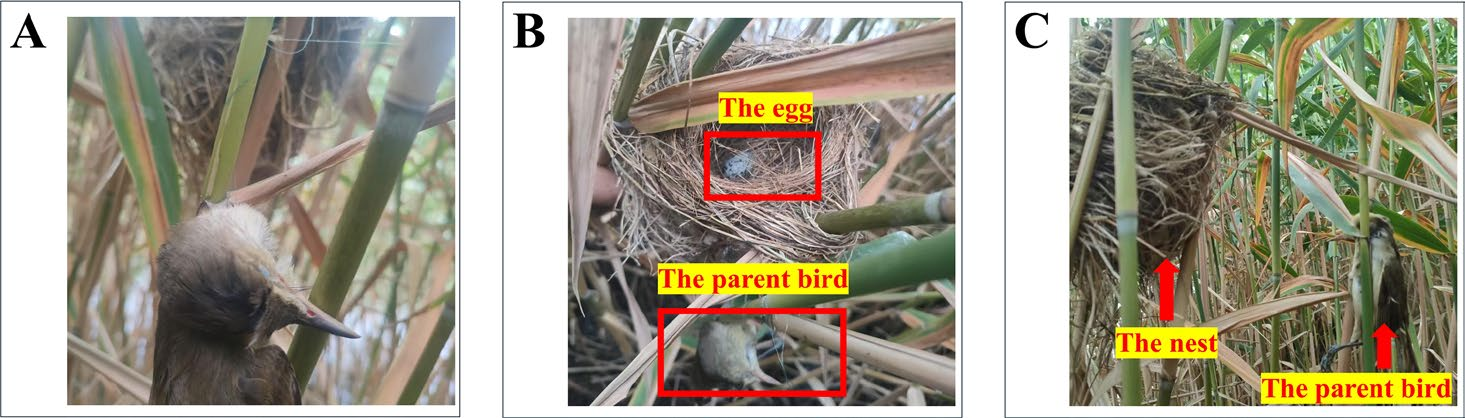
Entangled Oriental Reed Warbler and nest position. (A) Entangled Oriental Reed Warbler parent bird. (B) Parent bird and egg in the nest. (C) Relative position of the bird and the nest.

**FIGURE 2:**
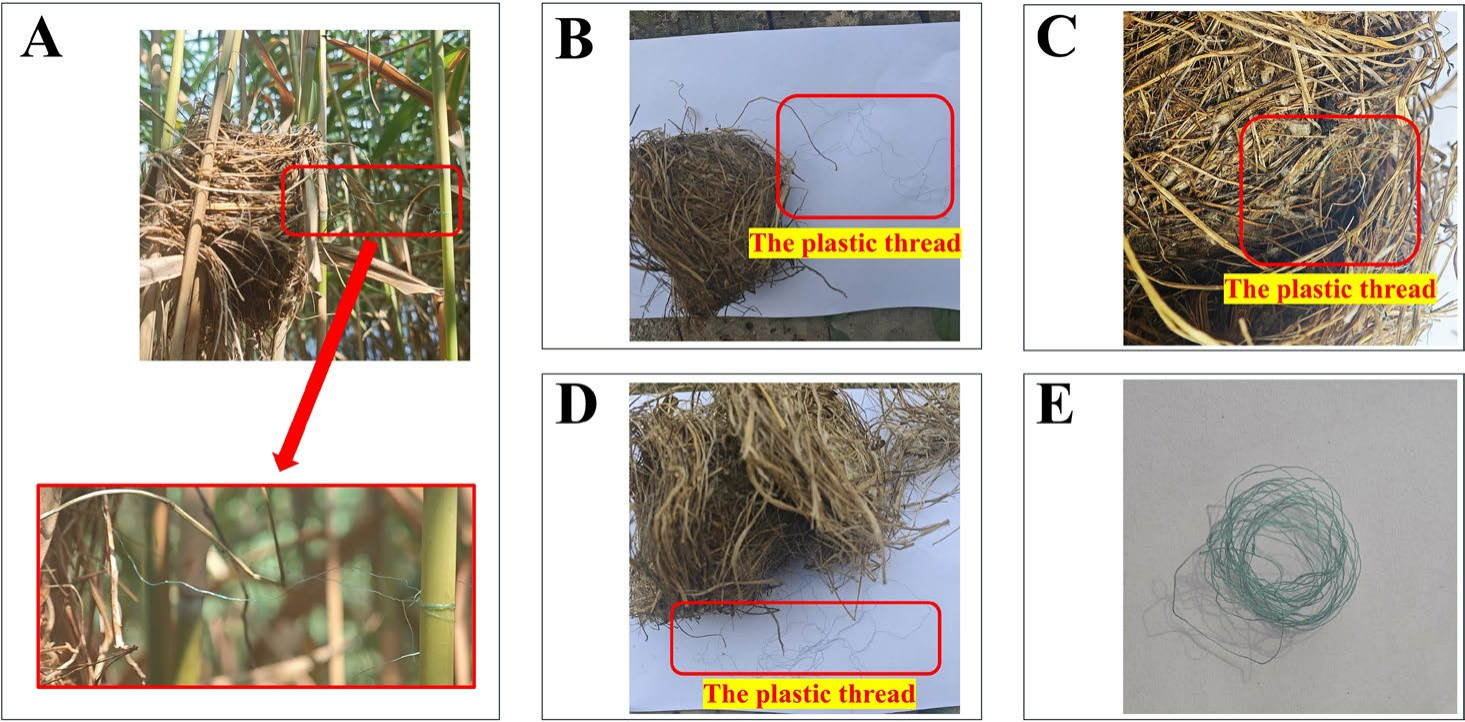
Nest of the entangled Oriental Reed Warbler. (A) Plastic string between the nest and reed stalk. (B) Complete nest. (C) Plastic string inside the nest. (D) Nest after dismantling. (E) Plastic string extracted from the nest.

During a systematic search for Oriental Reed Warbler breeding nests in the Baiyangdian Wetland at around 7:00 AM on June 28, 2025, an adult Oriental Reed Warbler was incidentally found dead due to entanglement in plastic string within a nest (Figure 1A), with only one egg present (Figure 1B). The bird was approximately 10 cm from the nest on a reed stalk (Figure 1C). Preliminary assessment indicated death by entanglement from plastic string in the nest. Further observation revealed that one side of the plastic string had been woven into the nest cavity by the parent bird, whereas the other side was entangled around an adjacent reed (Figure 2A). Such a configuration transformed the nest into a fixed trapping device, whereby the parent bird could become entangled during activity near the nest.

**FIGURE 3:**
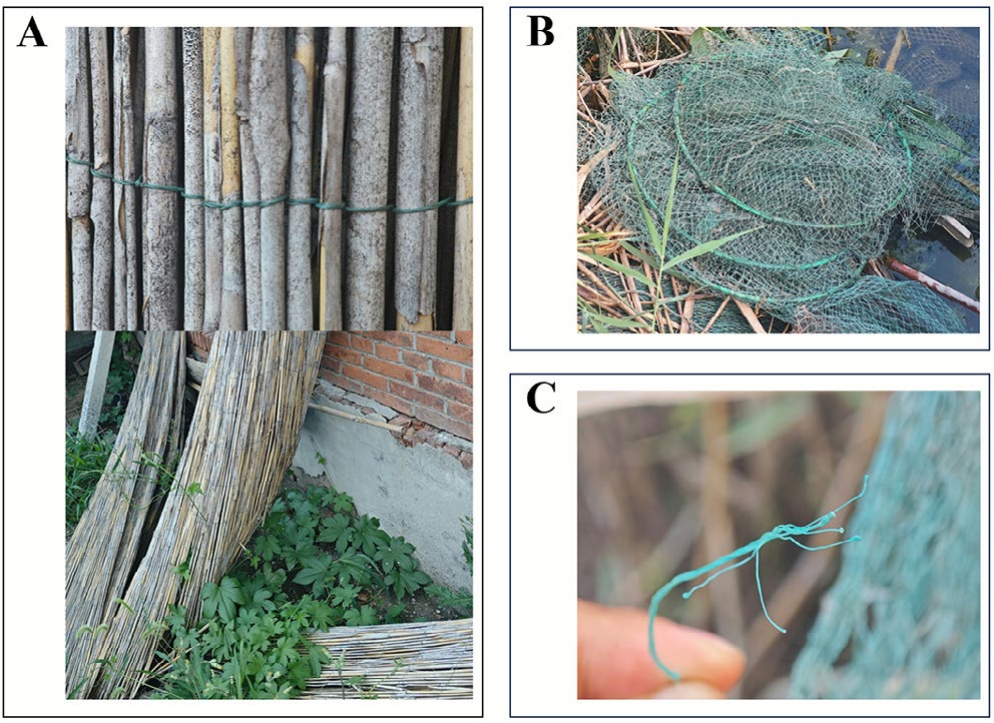
Common sources of plastic string surrounding the villages. (A) Plastic string used for weaving reed products. (B) Fish traps deployed at the water's edge. (C) Plastic string used to weave fish traps.

An Oriental Reed Warbler was observed maintaining vigilance near the nest; however, when the nest was revisited on July 5th, no additional new eggs were recorded, indicating that the other parent had abandoned the nest, resulting in reproductive failure. As no new eggs were laid in the nest and no parental incubation was observed, we infer based on this phenomenon that the individual that died from entanglement was likely the female. Subsequently, and without impacting local bird breeding activities, the nest was removed and dismantled, revealing that a plastic string had been woven into the inner layer of the nest (Figure 2B–D), with a total length of approximately 161 cm and a diameter of 0.13 mm (Figure 2E). Based on field investigations in the Baiyangdian area and visual inspection of this linear material, it is inferred that the string most likely originates from woven materials such as fishing gear or reed products and could also come from agricultural or packaging materials in the environment (Figure 3A–C).

## 3 Discussion

Numerous studies have reported cases of avian mortality due to entanglement; however, the birds involved in such studies are predominantly medium to large species (mostly seabirds) and nestlings (Costa et al. 2020; Janic et al. 2023; Parker and Blomme 2007; Heinze et al. 2025; Restani 2023; Votier et al. 2011; Ryan 2018; Mallet et al. 2020). Small‐bodied bird species are more likely to survive in areas with high anthropogenic disturbance, implying that their habitats would contain higher densities of artificial materials (Chen et al. 2024; Neate‐Clegg et al. 2023). However, only very few reports have addressed small passerine parent bird mortality due to artificial string entanglement (Antczak et al. 2010; Collins and Johnson 1982; Winiewicz et al. 2025). The present study reports a case of an Oriental Reed Warbler parent bird being entangled to death by plastic string in nesting material, leading to breeding failure, providing direct evidence of the hazards that artificial nesting materials pose to avian breeding and survival.

The Oriental Reed Warbler breeding process highly depends on biparental participation to complete egg incubation, nestling rearing, and nest defense (Ma and Liang 2021; Wang et al. 2013). Therefore, abandonment or death of one parent often indicates breeding failure (Kempenaers 2022; Li et al. 2015; Santema and Kempenaers 2018). In the present case, the nest was abandoned after one parent bird died, possibly related to the inability of a single parent to independently complete reproductive investment. The typical clutch size of Oriental Reed Warblers in the region is 4–5 eggs; the breeding nest contained only one egg, indicating an earlier stage of reproduction with lower overall offspring value, making nest abandonment more likely for the parent bird. According to parental investment theory, as breeding progresses toward later stages, offspring become increasingly valuable, parent birds invest more resources, and abandonment becomes less likely (Shew et al. 2016; Thünken et al. 2010).

Birds exhibit a certain degree of adaptability to the gradually increasing artificial nesting materials in the environment. Using such artificial nesting materials could reduce the difficulty of finding nesting materials for birds and accelerate nest‐building (Sheard et al. 2024). Certain types of artificial nesting materials can also increase nest stability (Antczak et al. 2010). Additionally, some artificial nesting materials (such as cigarette butts) can temporarily repel nest parasites (Suárez‐Rodríguez et al. 2013). However, the hazards of artificial nesting materials to birds remain significant. For example, plastic is a widely used artificial nesting material by wild birds, and avian contact with plastic nesting materials may lead to ingestion of toxic microplastics or entanglement mortality (Janic et al. 2023). Oriental Reed Warbler nests are primarily woven from reed materials; however, they also adaptively obtain artificial material products from the environment. The reed wetlands on which Oriental Reed Warblers highly depend also experience high intensities and frequencies of anthropogenic activities such as fishing, with a relatively high probability of abandonment of plastic products such as fishing nets and fishing lines, which persist and accumulate in the environment over extended periods. Such conditions further increase the use of artificial materials by the Oriental Reed Warbler (Xiong and Lu 2013). In the present study, the authors observed that the mode of deployment of the plastic string by the Oriental Reed Warbler established a trap‐like structure, possibly due to unfamiliarity of the individual with the properties of novel artificial materials such as fishing lines, leading to the death of the parent bird and breeding failure of the nest.

In summary, the present study reports a case of an Oriental Reed Warbler parent bird killed by plastic string in its nesting material, subsequently causing breeding failure, providing direct evidence of plastic hazards to avian reproduction and survival. As urbanization accelerates, the prevalence and harms of plastic products continue to grow, representing a major threat to wildlife survival. Future research should undertake more extensive investigations on the hazards to wildlife and their adaptive responses. In addition, appropriate measures should be adopted to minimize harm from plastics, such as cleaning plastic products from the environment and developing degradable and environment‐friendly materials.

## Author Contributions

**Haijie Zhang:** data curation (equal), formal analysis (equal), investigation (lead), writing – original draft (equal). **Yufeng Liu:** data curation (equal), formal analysis (equal), funding acquisition (equal). **Yingying Wang:** formal analysis (equal). **Gang Fu:** formal analysis (equal). **Laikun Ma:** conceptualization (lead), funding acquisition (equal), methodology (lead), writing – original draft (equal), writing – review and editing (equal). **Jianxin Dong:** writing – review and editing (equal).

## Funding

This work was funded by the National Natural Science Foundation of China (32101242 to L.M.), Science Research Project of Hebei Education Department (QN2025226 to L.M.), Project of Hebei Minzu Normal University (QN2024001 to L.M.) and the Science and Technology Project of Hebei Academy of Sciences (25A10 to Y.L.).

## Ethics Statement

The experiments reported here comply with the current laws of China. Fieldwork was carried out under permission from Baiyangdian Nature Reserve.

## Conflicts of Interest

The authors declare no conflicts of interest.

## Supporting information


**Data S1:** ece373009‐sup‐0001‐DataS1.xlsx.

## Data Availability

The data related to this study has been submitted simultaneously as Supporting Information.
